# Toxicity and Detoxification Enzyme Inhibition in the Two-Spotted Spider Mite (*Tetranychus urticae* Koch) by *Artemisia annua* L. Essential Oil and Its Major Monoterpenoids

**DOI:** 10.3390/insects16080811

**Published:** 2025-08-05

**Authors:** Fatemeh Nasr Azadani, Jalal Jalali Sendi, Asgar Ebadollahi, Roya Azizi, William N. Setzer

**Affiliations:** 1 Department of Plant Protection, Faculty of Agricultural Sciences, University of Guilan, Rasht 416351314, Iran; nsabaaa204@yahoo.com (F.N.A.); roya.aziizii1395@yahoo.com (R.A.); 2 Department of Plant Sciences, Moghan College of Agriculture and Natural Resources, University of Mohaghegh Ardabili, Ardabil 5697194781, Iran; 3 Department of Chemistry, University of Alabama in Huntsville, Huntsville, AL 35899, USA; 4 Aromatic Plant Research Center, 230 N 1200 E, Suite 100, Lehi, UT 84043, USA

**Keywords:** biochemical disturbances, botanical pesticides, 1,8-cineole, camphor, fumigant toxicity

## Abstract

The two-spotted spider mite, *Tetranychus urticae*, is one of the polyphagous pests of several crops and forestry, resistant to numerous conventional chemicals. Due to the negative side effects of harmful chemical pesticides, such as environmental pollution, and risks to human health, the introduction of effective and low-risk alternatives is essential. The promising pesticidal effects of essential oils (EOs) isolated from *Artemisia annua* have been documented in recent studies. In the present study, the acaricidal effects of an *A. annua* EO, along with its two dominant monoterpenoids, 1,8-cineole and camphor, were investigated against adults of *T. urticae*. *Artemisia annua* EO, 1,8-cineole, and camphor, with 24 h-LC_50_ values of 0.289, 0.533, and 0.64 µL/L air, respectively, had significant toxicity by fumigation against *T. urticae* adults. Along with lethality, *A. annua* EO and monoterpenoids had significant inhibitory effects on the activity of detoxifying enzymes, including α- and β-esterases, glutathione S-transferases, and cytochrome P-450 monooxygenase. According to the findings of the present study, *A. annua* EO and its two dominant monoterpenoids, 1,8-cineole and camphor, with significant toxicity and inhibitory effects on detoxifying enzymes, can be introduced as available, effective, and eco-friendly acaricides in the management of *T. urticae*.

## Simple Summary

Due to the negative side effects associated with harmful chemical pesticides, such as environmental pollution and risks to human health, it is essential to introduce effective and low-risk alternatives. This study investigated the acaricidal effects of an essential oil (EO) from *Artemisia annua* L., along with its two main monoterpenoids, 1,8-cineole and camphor, against adult *Tetranychus urticae* Koch. *Artemisia annua* EO and monoterpenoids exhibited significant toxicity against *T. urticae*. In addition to causing lethality, the EO and its monoterpenoids substantially inhibited the activity of detoxifying enzymes, including α- and β-esterases, glutathione S-transferases, and cytochrome P-450 monooxygenase. The results revealed that *A. annua* EO and 1,8-cineole and camphor demonstrate notable toxicity and inhibitory effects on detoxifying enzymes of *T. urticae*, identifying them as potential biorational pesticides.

## 1. Introduction

The two-spotted spider mite [*Tetranychus urticae* Koch (Acariformes, Tetranychidae)], a widely distributed species particularly in Asia, Europe, and North and South America, is one of the damaging pests of economically important crops in both outdoor crops and greenhouses [1]. Indeed, as a phytophagous pest, *T*. *urticae* has more than 3800 host plants around the world [2]. Larvae, nymphs, and adults of *T*. *urticae* feed on leaves, preferentially on lower surfaces, causing direct and indirect damage from the necrosis of young leaves and yellowing and reduction in the chlorophyll content of leaves leading to decline of the photosynthetic ability of the plant [3]. *Tetranychus urticae* damage can be very severe, especially in dry seasons, where a 40–60% reduction in soybean yield in field conditions has been reported [4].

Chemical pesticides are widely employed to protect plants from various arthropod pests such as *T*. *urticae*. Today, it is more obvious than ever that *T. urticae*, with parthenogenetic reproduction, high fecundity, and short life cycle, has an extraordinary ability to evolve resistance to several conventional pesticides, including abamectin, pyrethroids, and organophosphates [5,6]. Generally, due to side effects caused by synthetic pesticides, including the development of pest resistance, threat to human health, and environmental contamination [7], the introduction of eco-friendly efficient agents for managing detrimental pests is necessary.

Plant-derived essential oils (EOs), exhibiting low mammalian toxicity compared to synthetic chemicals, high biodegradability, and at the same time showing promising controlling potential against wide varieties of insects and mites, have been emphasized as biopesticides [8,9]. The promising pesticidal potential of EOs isolated from sweet wormwood [*Artemisia annua* L. (Asterales: Asteraceae)] against several destructive arthropod pests of stored products, crops, orchards, and forest trees was reported in recent studies. For example, the susceptibility of the elm leaf beetle [*Xanthogaleruca luteola* (Müller)], the red flour beetle [*Tribolium castaneum* (Herbst)], the lesser mulberry pyralid (*Glyphodes pyloalis* Walker), and the fall webworm [*Hyphantria cunea* (Drury)] to *A. annua* EO was documented [10,11,12,13]. Indeed, the *A. annua* EO showed diverse pesticidal effects from lethality to physiological disorders against several pests [14,15,16]. For instance, *A. annua* EO caused a significant reduction in activity detoxifying enzymes, including α- and β-esterase and glutathione S-transferase (GST), of the larvae of *G. pyloalis* [16].

Chemical composition analyses indicated terpenes are the main identified groups in the *A. annua* EO [14,16]. For example, 1,8-cineole, camphor, pinocarvone, α-pinene, and δ-3-carene were identified as dominant compounds in the EO of vegetative growth stage of *A. annua* [14]. In another study, camphor, artemisia ketone, β-selinene, pinocarvone, 1,8-cineole, and α-pinene were dominant in the floral EO of *A. annua* [16]. According to recent studies, terpenic compounds, particularly monoterpene hydrocarbons and oxygenated monoterpenoids, can indicate considerable pesticidal properties [17,18,19]. For example, along with contact toxicity on third instar larvae of *H. cunea*, camphor and 1,8-cineole caused significant inhibition on the activity of detoxifying esterases and glutathione S-transferase enzymes [13].

*Artemisia annua* EO, along with its major components, camphor and 1,8-cineole, is generally considered to have low toxicity to beneficial organisms like predators and pollinators [20,21,22]. For example, Seixas et al. [20] revealed that *A. annua* EO was selective for the predator fire ant (*Solenopsis saevissima* (Smith)) and pollinator jataí bee (*Tetragonisca angustula* (Latreille)) adults, while causing high mortality in the melonworm (*Diaphania hyalinata* (L.)) larvae.

The main objective of the present study is to investigate the pesticidal effects of *A. annua* EO against the adults of cosmopolitan and polyphagous spider mite *T. urticae*, in which the fumigant toxicity and inhibitory effects on the activity of detoxifying enzymes, including esterases, glutathione S-transferase, and cytochrome P450 monooxygenases, were assessed. Additionally, the pesticidal effects of the two dominant monoterpenoids in *A. annua* EO, 1,8-cineole and camphor, were evaluated.

## 2. Materials and Methods

### 2.1. Cultivation of Cowpea

With fast growth, easy cultivation in small spaces, and long life periods, the cowpea (*Vigna unguiculata* (L.) Walp. (Fabaceae)) is a suitable host plant for the rearing of *T. urticae* [23]. Cowpea seeds were kept in a wet towel for 2–3 d to stimulate their germination. Then, seeds were planted in the soil in the greenhouse of Faculty of Agricultural Sciences, University of Guilan, Iran, at a depth of 3 cm. To provide healthy and sufficient host plants, new seeds were regularly cultivated. The plants were checked daily, quickly removing any pests or pathogenic agents.

### 2.2. Mass Rearing and Synchronization of the Pest

*Tetranychus urticae* colonies were collected from rose plants (*Rosa* spp. (Rosaceae)) on the campus of the Faculty of Agricultural Sciences at the University of Guilan, which had no history of pesticide spraying. The colonies were transferred to the college’s greenhouse for the mass rearing of cowpeas.

To achieve a pure population of *T. urticae*, the leaves infected with the pest were moved from the greenhouse to the laboratory. The *T. urticae* were removed by a brush, placed on the cowpea-leaf disk, and transferred to the germinator at 26 ± 2 °C, 65 ± 5% RH, and under 16:8 light to dark conditions. These leaves were transferred to glass containers (14 cm diameter and 1.5 cm height) whose bottoms were covered with a layer of wet cotton, moistened and replaced with new leaves daily. The pure colonies of *T. urticae* were relocated by a brush on cowpeas in the greenhouse [24].

To synchronize the *T. urticae* adults, leaves containing adult mites (male and female) were separated from the colonies in the greenhouse, and 1000 adult mites were placed on cowpea-leaf disks, kept in the germinator at 26 ± 2 °C, 65 ± 5% RH, and under 16:8 light to dark conditions. After 48 h, the adult mites were removed from the leaf disk using a fine brush. Synchronized adult mites were attained from the hatched eggs after 10–12 d, which were used for bioassays [25].

### 2.3. Essential Oil and Compounds

The leaves of the *A. annua* were collected from the campus of Guilan University and dried in the shade within a week. After powdering the plant samples (Model HR2106 Philips, Amsterdam, The Netherlands), EO extraction was performed using a Clevenger apparatus (J3230, Sina glass, Tehran, Iran) and water distillation method. Fifty grams of the powder was poured into a Clevenger flask, 650 mL of distilled water was then added, and after soaking the plant in distilled water for 24 h, the EO extraction was performed within 4 h [14]. Sodium sulfate was used to remove water from extracted EO. The obtained EO was transferred to vials, which were covered with aluminum foil, and then stored in the refrigerator at 4 °C. 1,8-Cineole and camphor were purchased from Sigma Aldridge Company (St. Louis, MO, USA).

### 2.4. Essential Oil Composition

The chemical composition of the essential oil was characterized using gas chromatography–mass spectrometry (GC-MS) (Agilent Technologies 7890B GC system coupled with an Agilent Technologies 5977A MS detector, Santa Clara, CA, USA). Capillary column was HP-5MS (30 m × 0.25 mm, 0.25 µm film thickness), and helium was used as the carrier gas at a constant flow rate of 1 mL/min. The GC oven temperature program was initiated at 50 °C (held for 1 min), followed by a gradual increase at 6 °C/min until reaching 290 °C, resulting in a total analysis time of 50 min. Prior to analysis, a 10% (*v*/*v*) dilution of essential oil in methanol was prepared, and 1 µL of the solution was injected in splitless mode. Mass spectra were acquired in electron impact (EI) mode at 70 eV ionization energy. Retention indices were calculated based on a homologous series of *n*-alkanes (C_8_–C_20_) analyzed under identical conditions. The constituent compounds were recognized by comparison of their mass spectral fragmentation patterns and retention indices with the established literature [26,27,28].

### 2.5. Fumigant Toxicity

A 6 cm cowpea-leaf disk was placed on wet cotton in a Petri dish (6 cm), and 10 adult mites were transferred to each disk. The Petri dish was placed inside a transparent plastic fumigation chamber (1000 mL volume). Five concentrations of *A. annua* EO, 1,8-cineole, and camphor (0.125, 0.25, 0.5, 1, and 2 µL/L air) were selected based on preliminary tests that caused 10% to 90% mite mortality. The filter papers were treated with the considered amounts of the three compounds (0.125, 0.25, 0.5, 1, and 2 μL) using a digital microdispenser (Drummond Digital Microdispenser, Drummond Scientific Company, Broomall, PA, USA) and then transferred into the fumigant chambers closed air-tightly. Pest mortality was recorded after 24 and 48 h. The experiments were conducted in a completely randomized design with four replications. All steps were performed for the negative control group, except for the treatment of filter paper with EO and compounds [29].

### 2.6. Determination of Enzyme Activity

Adults of *T. urticae* treated with *A. annua* EO, 1,8-cineole, and camphor with LC_30_ and LC_50_ and the number of live mites were collected after 24 h of treatment. The whole body of treated adults (100 in number) was homogenized manually in phosphate buffer (1:1 *w*/*v* at pH 7) and centrifuged (Eppendorf 5417R centrifuge, Hamburg, Germany) at 13,000× *g* for 20 min at 4 °C. The supernatant liquid was used as the source of enzymes in three replications for each biochemical experiment, and the absorbance rate was read at specified wavelength (Epoch 2 Microplate reader, BioTek, Winooski, VT, USA).

#### 2.6.1. General Esterase (EST)

General esterases were measured using two substrates including α-naphthyl acetate (α-NA) and β-naphthyl acetate (β-NA) according to the method of Han et al. [30]. Briefly, 10 μL of each substrate (10 mM) was added separately to 30 μL of universal buffer (pH 7) and 5 μL of fast blue RR salt (1 mM) before the addition of 20 μL of enzyme solution. Incubation continued for 5 min, and then the absorbance rate was read at 450 nm wavelengths.

#### 2.6.2. Glutathione S-Transferase (GST)

Glutathione S-transferase activity was performed according to Oppenorth [31] using the reagents CDNB (1-chloro-2,4-dinitrobenzene, 20 mM) and DCNB (1,2-dichloro-4-nitrobenzene, 20 mM). The reaction solution contained 20 μL of enzyme solution, 30 μL of universal buffer (pH 7), 20 μL CDNB, and 20 μL DCNB. Activity of GSTs was recorded with the use of a microplate reader at 340 nm at 30 s intervals for 3 min.

#### 2.6.3. Cytochrome P450 Monooxygenase Activity

The whole body of the treated adults of *T. urticae* was homogenized in sodium phosphate buffer (0.04 mM, pH 7) and centrifuged at 13,000× *g* and 4 °C for 20 min, and the supernatant was used as an enzyme extract [32]. Cytochrome P450 monooxygenase enzyme activity was measured using TMB substrate (3,3′,5,5′ tetramethylbenzidine dihydrochloride) based on the method of Martin et al. [33]. The reaction mixture included 50 μL sodium phosphate buffer (100 mM, pH 7.2), 50 μL enzyme extract, and 150 μL TMB. Then, 25 μL hydrogen peroxide (3%) was added, and after 30 min of incubation at room temperature, the absorbance was recorded at 630 nm.

### 2.7. Statistical Analysis

The Lethal Concentration (LC_30_, LC_50_, and LC_90_ after 24 and 48 h) and regression line information were estimated through Probit analysis using Polo-Plus software (version 2.0) [34]. Data from enzymatic bioassays were analyzed by one-way analysis of variance (ANOVA) and the means compared using Tukey’s multiple range tests at a 5% level. Analysis of the effect of concentration and time on pest mortality (two-factor test) was performed in SAS 9.1 software [35]. Data were sorted and graphs created using Excel 2021.

## 3. Results

### 3.1. Chemical Composition of Essential Oil

The chemical composition of the essential oil obtained from leaves of *A*. *annua* is presented in Table 1. Along with camphor (18.7%) and 1,8-cineole (7.5%), α-Pinene (7.5%), (*E*)-β-caryophyllene (6.4%), β-selinene (5.9%), artemisia ketone (4.2%), germacrene D (3.3%), and β-pinene (3.1%) were detected as the major compounds, all of which are terpenes. Almost 88% of recognized compounds were from terpenes, in which oxygenated monoterpenoids were present in high amounts (40.8%). However, the monoterpene hydrocarbons (13.5%), sesquiterpene hydrocarbons (21.6%), and oxygenated sesquiterpenoids (11.6) had high quantity in the *A. annua* EO.

### 3.2. Fumigant Toxicity

**Figure 1:**
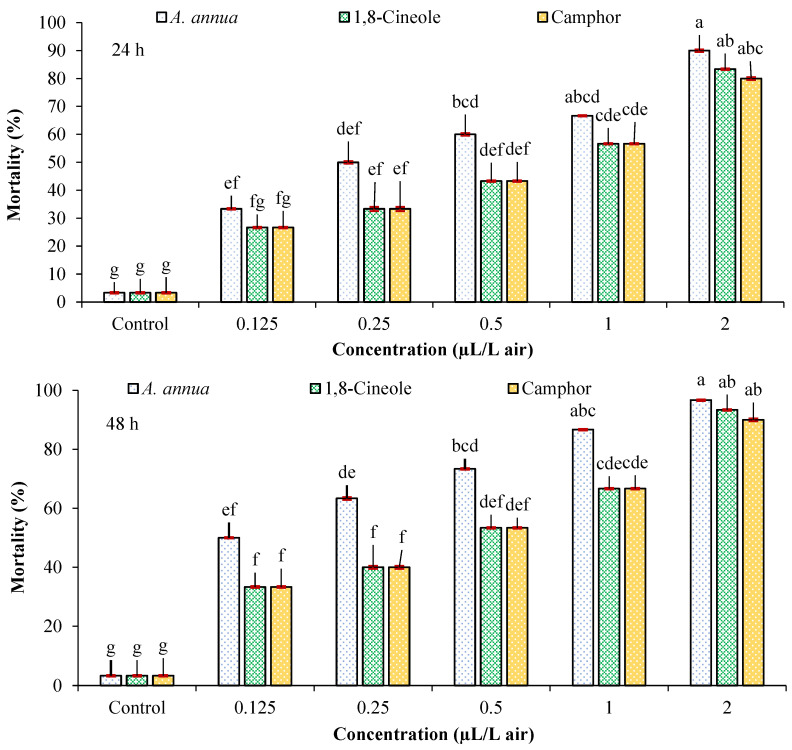
The mortality (mean ± SE) of *Tetranychus urticae* adults treated with *Artemisia annua* essential oil and the monoterpenoids camphor and 1,8-cineole after 24 h (F = 30.07; *df* = 17, 53; *p* < 0.0001) and 48 h (F = 56.63; *df* = 17, 53; *p* < 0.0001). Columns sharing the same letters are not significantly different according to Tukey’s tests (*p* ≤ 0.05).

Results of the Probit analysis of data obtained from the fumigant toxicity of *A*. *annua* EO, 1,8-cineole, and camphor against *T*. *urticae* adults are shown in Table 2 and Figure 1. The values of LC_30_, LC_50,_ and LC_90_ for all three compounds after 24 and 48 h, 95% confidence limits, and the slope of the regression line were estimated. The results of the analysis of variance for the data obtained from the fumigant toxicity of *A*. *annua* EO, 1,8-cineole, and camphor, respectively, showed that the concentrations used (F = 43.03, *df* = 4, and *p* < 0.0001), (F = 45.58, *df* = 4, and *p* < 0.0001), (F = 46.24, *df* = 4, and *p* < 0.0001) and the exposure times (F = 28.47, *df* = 1, and *p* < 0.0001), (F = 7.20, *df* = 1, and *p* = 0.0143), (F = 3.76, *df* = 1, and *p* = 0.0666) had a significant effect on the mortality of the *T*. *urticae*, but their interaction effects (F = 0.38, *df* = 4, and *p* = 0.8186), (F = 0.08, *df* = 4, and *p* = 0.9890), (F = 0.24, *df* = 4, and *p* = 0.9151) were not significant. The highest toxicity is related to the EO of *A*. *annua* with a 48 h LC_50_ of 0.147 µL/L air, and the lowest is related to the camphor with a 24 h LC_50_ value of 0.640 µL/L air. Also, the LC_90_ values show the high potential of *A*. *annua* EO, 1,8-cineole, and camphor terpene compounds in controlling the two-spotted spider mite. According to high R^2^ values of all tested agents, the mortality of *T*. *urticae* has a positive and direct relation with tested concentrations of EO and monoterpenoids. Although the overlapping can be detected among confidence limits, based on high relative potency, *A*. *annua* EO was more toxic than both monoterpenoids.

### 3.3. Effects on Detoxifying Enzymes Activity

**Figure 2:**
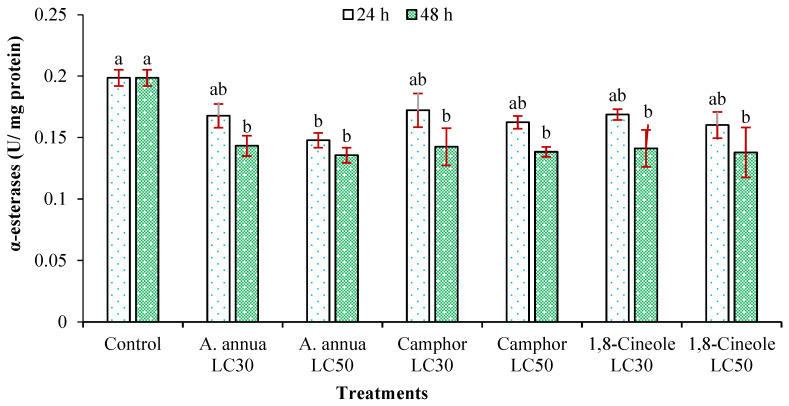
The activity of α-esterases (mean ± SE) of *Tetranychus urticae* adults treated with *Artemisia annua* essential oil and the monoterpenoids camphor and 1,8-cineole after 24 (F = 3.09; *df* = 6, 20; *p* = 0.0384) and 48 h (F = 3.39; *df* = 6, 20; *p* = 0.0279). Treatment columns sharing the same letter are not significantly different according to Tukey’s tests (*p* ≤ 0.05).

**Figure 3:**
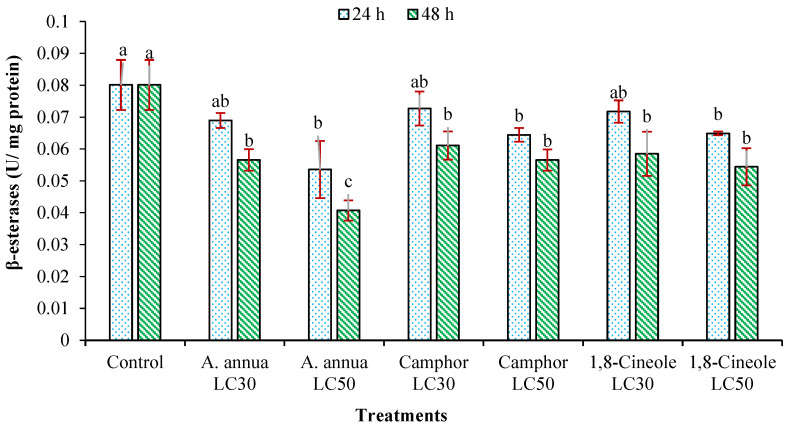
The activity of β-esterases (mean ± SE) of *Tetranychus urticae* adults treated with *Artemisia annua* essential oil and the monoterpenoids camphor and 1,8-cineole after 24 (F = 2.51; *df* = 6, 20; *p* = 0.0732) and 48 h (F = 4.83; *df* = 6, 20; *p* = 0.0072). Treatment columns sharing the same letter are not significantly different according to Tukey’s tests (*p* ≤ 0.05).

Variations in the activity of α- and β-esterases of *T*. *urticae* adults treated with *A*. *annua* EO and the monoterpenoids camphor and 1,8-cineole are shown in Figure 2 and Figure 3. The reduction in the number of α- and β-esterase enzymes of *T*. *urticae* adults affected by LC_30_ of all tested agents had no significant difference compared to the control group. However, the LC_50_ of *A*. *annua* EO and 1,8-cineole decreased the activity of both α- and β-esterases within 48 h. Monoterpenoid camphor was only able to significantly reduce the activity of α-esterase enzyme after 48 h compared to the control group.

**Figure 4:**
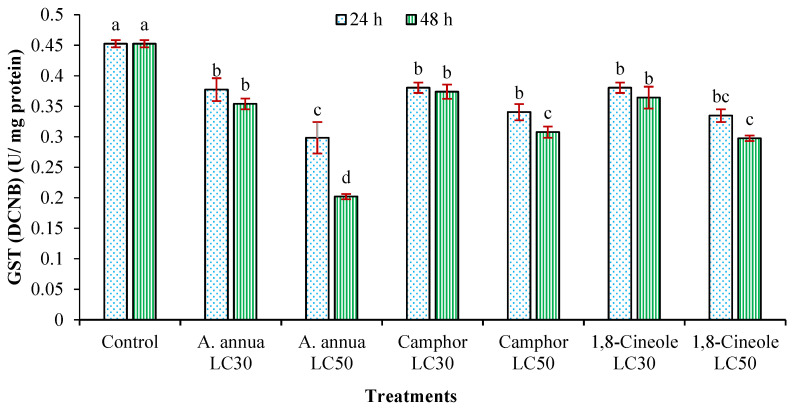
The activity of glutathione S-transferase (GST) (mean ± SE) of *Tetranychus urticae* adults, in reagent DCNB, treated with *Artemisia annua* essential oil and the monoterpenoids camphor and 1,8-cineole after 24 (F = 11.2; *df* = 6, 20; *p* = 0.0001) and 48 h (F = 108.25; *df* = 6, 20; *p* = 0.0001). Treatment columns sharing the same letter are not significantly different according to Tukey’s tests (*p* ≤ 0.05).

**Figure 5:**
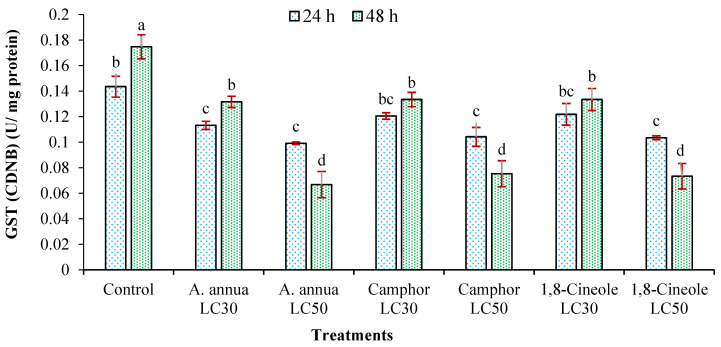
The activity of glutathione S-transferase (GST) (mean ± SE) of *Tetranychus urticae* adults, in reagent CDNB, treated with *Artemisia annua* essential oil and the monoterpenoids camphor and 1,8-cineole after 24 (F = 7.58; *df* = 6, 20; *p* = 0.0009) and 48 h (F = 22.55; *df* = 6, 20; *p* = 0.0001). Treatment columns sharing the same letter are not significantly different according to Tukey’s tests (mean ± SEM) (*p* ≤ 0.05).

According to Figure 4 and Figure 5, the amount of GST was decreased in both reagents CDNB and DCNB by LC_30_ and LC_50_ of *A*. *annua* EO after 24 and 48 h exposure times. Although the decrease in the amount of GST by LC_30_ of camphor and 1,8-cineole was not significant after 24 h, by the increasing time to 48 h and utilizing concentration to LC_50_, significant decreases were realized compared to the control group.

**Figure 6:**
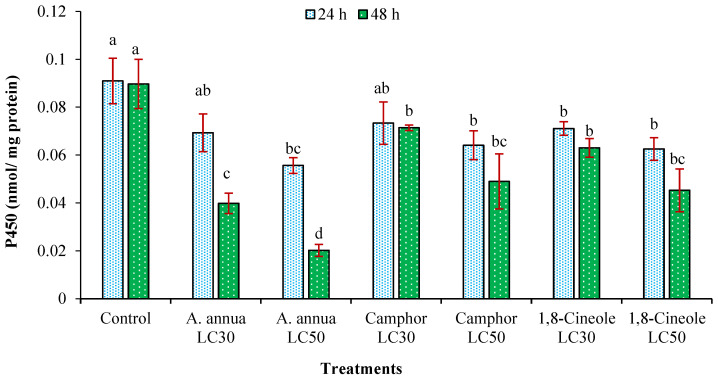
The activity of cytochrome P450 monooxygenase (CYP450) (mean ± SE) of *Tetranychus urticae* adults treated with *Artemisia annua* essential oil and the monoterpenoids camphor and 1,8-cineole after 24 (F = 2.82; *df* = 6, 20; *p* = 0.0513) and 48 h (F = 10.1; *df* = 6, 20; *p* = 0.0002). Treatment columns sharing the same letter are not significantly different according to Tukey’s tests (*p* ≤ 0.05).

Both LC_30_ and LC_50_ of *A*. *annua* EO were able to reduce the activity of cytochrome P450 monooxygenase of *T*. *urticae* after 48 h. The activity of this enzyme was also significantly decreased by LC_50_ of camphor and 1,8-cineole after 48 h (Figure 6).

## 4. Discussion

According to the results of the present study, EO isolated from *A. annua*, with an LC_50_ value of 0.289 and 0.147 µL/L air after 24 and 48 h, respectively, has high fumigant toxicity against the adults of *T. urticae*. The susceptibility of *T. urticae* to plant-derived EOs was also demonstrated in previous studies. For example, the EOs of *Achillea millefolium* L. (LC_50_ = 1.80 μL/L), *Eucalyptus oleosa* F. Muell. ex Miq. (LC_50_ = 2.42 μL/L), *E. torquata* Luehm. (LC_50_ = 3.59 μL/L), *Thymus eriocalyx* (Ronniger) Jalas (LC_50_ = 0.82 μL/L), and *Thymus kotschyanus* Boiss & Hohen (LC_50_ = 1.77 μL/L) showed considerable fumigant toxicity against *T. urticae* [29,36,37]. Regarding EOs of the *Artemisia* genus, Esmaeily et al. [38] revealed that *A. annua* EO with an LC_50_ value of 4.14 µL/80 mL air was toxic to the adults of *T. urticae*. Furthermore, they also indicated significant decreases in fecundity, generation time, adult longevity, net reproductive rate (*R*_0_), and intrinsic rate of increase (*r*), finite rate of increase (λ) of *T. urticae* by the EO of *A. annua*. The results of the aforementioned studies, in line with the findings of the present study, indicate the susceptibility of *T. urticae* to EOs. However, the differences in the LC_50_ values can be caused by different species of EOs and, accordingly, their different chemical compositions.

Monoterpenoids (C_10_) are volatile and lipophilic natural agents that can rapidly penetrate the pest’s body [39]. There are several reports regarding the pesticidal potential of monoterpenoids 1,8-cineole (synonym: eucalyptol, C_10_H_18_O) and camphor (C_10_H_16_O), as main compounds in the *A. annua* EO, against detrimental arthropods. For example, contact toxicity against the larvae (24 h LD_50_ = 77.0 mg/L) and fumigant toxicity against the adults (24 h LC_50_ = 3.3 μL/L) of the diamondback moth, *Plutella xylostella* (L.) of 1,8-cineole were reported [40]. In another study, contact and fumigant toxicity (with 24 h LC_50_ of 11.3 μg/adult and 2.9 mg/L, respectively) of camphor against the adults of the cigarette beetle, *Lasioderma serricorne* (F.) were verified [41]. According to show findings, 1,8-cineole and camphor, with 24 h LC_50_ values of 0.533 and 0.640 µL/L, have promising toxicity against the adults of *T. urticae*. Differences in the LC_50_ values can be justified by different tested pests and experimental conditions. However, the above-mentioned results approved present findings regarding pesticidal potential of 1,8-cineole and camphor. It was also found that the pesticidal properties of *Artemisia* EOs are related to their compounds, specifically the terpenic ones [42,43]. For instance, Liu et al. [18] demonstrated that the fumigant toxicity and acetylcholine esterase inhibitory of *Artemisia nakaii* Pamp EO against larvae of the tobacco armyworm (*Spodoptera litura* (F.)) were related to its main compounds such as camphor and 1,8-cineole.

The chemical composition of *A. annua* EO has been investigated by several recent studies [14,44,45].

Although 1,8-cineole and camphor were dominant in the *A. annua* EO [16,46,47,48], other components, such as camphene, carvacrol, caryophyllene oxide, germacrene d, spathulenol, β-pinene, and pinocarvone could also be identified in the present study. For example, along with camphor (32.5–58.9%) and 1,8-cineole (13.7–17.8%), camphene (4.5–8.4%) was high in quantity in an *A. annua* EO from Tajikistan [48]. In another study, β-selinene (10.7%), pinocarvone (7.4%), α-pinene (5.9%), and caryophyllene oxide (5.4%) were reported as main components of an *A. annua* EO from Iran [16]. Significant differences were observed between the chemical composition of *A*. *annua* EO in the present study and those reported in previous research. For example, camphene was present at a higher concentration (4.5–8.4%) in the *A*. *annua* EO from Tajikistan [48] compared to our findings (2.1%). Similarly, the levels of β-selinene (5.9%) and caryophyllene oxide (1.4%) in the current study were lower than those reported by Oftadeh et al. [16] found in Iranian plants (10.7% and 5.4%, respectively). In general, the chemical profile of EOs is variable depending on several exogenous and endogenous factors such as geographical location, extraction methods, and growing conditions [48,49,50]. Furthermore, in addition to 1,8-cineole and camphor, *T. urticae* was susceptible to other EO compounds such as limonene, linalool, thymol, and α-pinene [49,50]. Accordingly, it can be concluded that although the fumigant toxicity of *A. annua* EO may be related to its main compounds such as 1,8-cineole and camphor, other compounds can also be effective.

Glutathione S-transferases, esterases, and cytochrome P450 monooxygenases are the main groups of detoxifying enzymes in arthropods, which play an important role in pest resistance against pesticides. Glutathione S-transferases (GSTs) catalyze the conjugation of reduced glutathione with electrophilic components of both external and internal origin, converting them into water-soluble, less toxic forms for detoxification [51]. Esterases, including α- and β-esterases as well as acetylcholinesterase, represent a large and diverse group of hydrolases. They can hydrolyze various compounds—particularly ester bonds—by reacting with water to produce corresponding acids and alcohols, thereby contributing to detoxification [52]. Cytochrome P-450 monooxygenases are responsible for catalyzing the oxidation of external- and internal-origin compounds, in which they catalyze an atom of molecular oxygen into the substrate, while another atom is reduced to water [53]. Increasing the activity and amount of these detoxifying enzymes is one of the important mechanisms for arthropod pests to become resistant to pesticides. The significant inhibition of the detoxifying enzyme activity of *Artemisia* EOs against arthropods was emphasized [54,55]. For example, the inhibition of glutathione S-transferase of *P. xylostella* larvae treated with *Artemisia lavandulaefolia* DC. EO was reported [41]. Recent findings indicated that *A. annua* EO can inhibit the activity of detoxifying enzymes. The activity of esterase and glutathione S-transferase enzymes of *G. pyloalis* larvae was significantly inhibited by the concentrations of 0.65% and 2.59 μL/L (for oral and fumigant toxicity, respectively) of *A. annua* EO [14].

According to the study of Mojarab-Mahboubkar et al. [13], treatment by a concentration of 177.08 μg/larva of *A. annua* EO caused a significant inhibition in the activity of α- and β-esterases and glutathione S-transferases of the larvae of *H. cunea*. The same results, regarding the inhibitory effects of *A. annua* EO on esterase and glutathione S-transferase enzymes, were also obtained in the present study but with another arthropod pest: *T. urticae*. Furthermore, it was demonstrated for the first time that *A. annua* EO at concentrations of 0.289 and 0.147 µL/L air (24 and 48 h) is able to prevent the activity of cytochrome P-450 monooxygenase in *T. urticae* adults. Additionally, the prevention of the activity of cytochrome P-450 monooxygenase, α- and β-esterases, and glutathione S-transferases of *T. urticae* adults by the monoterpenoids 1,8-cineole and camphor is among other worthy achievements of this study, indicating the pesticidal relationship between *A. annua* EO and its compounds. It can also be supported by the study of Mojarab-Mahboubkar et al. [13], in which 1,8-cineole and camphor reduced the activity of α- and β-esterases and glutathione S-transferases of the larvae of *H. cunea*.

## 5. Conclusions

According to the findings of the present study, *A. annua* EO has promising potential in the management of *T. urticae* based on its lethality and detoxifying enzyme inhibitory effects. The prevention of the activity of *T. urticae* detoxifying enzymes, including α- and β-esterases, glutathione S-transferases, and cytochrome P-450 monooxygenase, indicates that, along with high susceptibility of the pest, the chances of pest creating resistance against EO are low. Furthermore, *A. annua* is one of the highly distributed pasture plants in Iran, and it is possible to obtain high amounts of this EO. The fumigant toxicity and inhibitory effects on the detoxifying enzyme activity of *T. urticae* treated with monoterpenoids 1,8-cineole and camphor, recognized in *A. annua* EO in the previous studies, were also determined in this research. Despite the advantages of *A. annua* EO in the management of *T. urticae*, its effectiveness, availability, and eco-friendly features, its durability under environmental conditions is low. There are several methods based on controlled-release techniques which augment the stability and effectiveness of EOs, of which nano- and micro-encapsulation and/or preparing the nanoemulsion formulations of *A. annua* EO are documented in a recent study. It can be said that the pesticidal effects of *A. annua* EO are associated with their chemical compounds, particularly 1,8-cineole and camphor. It may also be concluded that the EOs with a high amount of 1,8-cineole and camphor can have pesticidal properties against *T. urticae*. However, the interaction of other compounds should be considered.

## Figures and Tables

**Table 1:** Chemical composition of the essential oil obtained from leaves of *Artemisia anuua*.

RI_calc_	RI_db_	Compound	%	RI_calc_	RI_db_	Compound	%
925	926	Tricyclene	0.1	1431	1432	β-Copaene	0.5
939	939	α-Pinene	7.5	1445	1451	*iso*-Germacrene D	0.2
953	950	Camphene	2.1	1455	1454	α-Humulene	0.7
978	979	β-Pinene	3.1	1478	1477	γ-Gurjunene	2.4
995	990	Myrcene	0.2	1485	1485	Germacrene D	3.3
1005	999	Yomogi alcohol	0.1	1493	1490	β-Selinene	5.9
1020	1025	p-Cymene	0.6	1500	1493	Capillene	2.9
1028	1031	1,8-Cineole	7.5	1516	1513	γ-Cadinene	0.2
1060	1062	Artemisia ketone	4.2	1524	1523	δ-Cadinene	0.4
1085	1083	Artemisia alcohol	0.4	1580	1578	Spathulenol	1.1
1102	1098	trans-Sabinene hydrate	0.3	1586	1583	Caryophyllene oxide	1.0
1138	1139	trans-Sabinol	1.5	1595	1594	Salvial-4(14)-en-1-one	0.2
1150	1146	Camphor	18.7	1602	1601	Helifolen-12-al B	0.5
1170	1164	Pinocarvone	2.4	1625	1630	Caryophylla-4(12),8(13)-dien-5α-ol	0.9
1172	1169	Borneol	0.9	1634	1636	cis- Cadin-4en-7-ol	0.9
1177	1177	Terpinen-4-ol	0.5	1638	1642	Caryophylla-4(12),8(13)-dien-5β-ol	1.2
1192	1188	α-Terpineol	0.2	1666	1669	ar-Turmerone	2.9
1199	1195	Myrtenol	1.0	1675	1669	14-Hydroxy-9-epi-(E)-caryophyllene	0.3
1218	1216	trans-Carveol	0.3	1690	1686	Germacra-4(15),5,10(14)-trien-1α-ol	1.6
1244	1243	Carvone	0.4	1701	1699	β-Turmerone	1.0
1287	1290	Thymol	0.7	1841	1841	Phytone	0.3
1298	1299	Carvacrol	1.5	2108	2109	Phytol	0.6
1336	1338	δ-Elemene	0.2	Monoterpene hydrocarbons			13.5
1376	1376	α-Copaene	1.4	Oxygenated monoterpenoids			40.8
1396	1395	Benzyl pentanoate	0.3	Sesquiterpene hydrocarbons			21.6
1390	1388	β-Cubebene	0.1	Oxygenated sesquiterpenoids			11.6
1398	1392	(Z)-Jasmone	0.1	Diterpenoids			0.6
1416	1419	(*E*)-β-Caryophyllene	6.4	Others			3.7
				Total identified			91.7

RI_calc_ = retention index determined with respect to a homologous series of n-alkanes on a HP-5 ms column; RI_db_ = retention index from the databases [26,27,28].

**Table 2:** Results of the Probit analysis of data obtained from the fumigant toxicity (24 h) of *Artemisia annua* L. essential oil, 1,8-cineole, and camphor against the adults of *Tetranychus urticae* Koch.

Tested Agent	Time (h)	Lethal Concentrations with 95% Confidence Limits (µL/L Air)			Slope ± SE	χ^2^ (*df* = 3)	*p* Value	RP	R^2^
		LC_30_	LC_50_	LC_90_					
*A. annua* essential oil	24	0.107 (0.032–0.186)	0.289 (0.158–0.433)	3.266 (1.640–16.087)	1.217 ± 0.268	1.741	0.628	2.215	0.926
	48	0.066 (0.019–0.118)	0.147 (0.069–0.221)	1.027 (0.660–2.479)	1.518 ± 0.319	2.438	0.487	4.354	0.987
1,8-Cineole	24	0.199 (0.087–0.310)	0.533 (0.349–0.836)	5.890 (2.642–37.458)	1.228 ± 0.262	2.170	0.538	1.201	0.913
	48	0.138 (0.054–0.223)	0.351 (0.216–0.514)	3.427 (1.763–14.679)	1.296 ± 0.268	2.090	0.554	1.823	0.919
Camphor	24	0.241 (0.115–0.367)	0.640 (0.428–1.041)	6.939 (3.023–47.308)	1.238 ± 0.263	0.748	0.862	1.000	0.971
	48	0.158 (0.056–0.260)	0.446 (0.274–0.695)	5.665 (2.482–41.601)	1.161 ± 0.260	0.283	0.936	1.435	0.987

RP: Relative Potency = the largest LC_50_/LC_50_ of another agent, χ^2^: Chi-square value, and *df*: degrees of freedom.

## Data Availability

The original contributions presented in this study are included in the article. Further inquiries can be directed to the corresponding authors.

## Abbreviations

The following abbreviations are used in this manuscript:

CDNB	1-chloro-2,4-dinitrobenzene
CYP450	cytochrome P450 monooxygenase
DCNB	1,2-Dichloro-4-nitrobenzene
*df*	Degrees of freedom
EO	Essential oil
EOs	Essential oils
ESTs	General esterases
GST	Glutathione S-transferase
LC_30_	Lethal Concentration to kill 30% of tested insects
LC_50_	Lethal Concentration to kill 50% of tested insects
LC_90_	Lethal Concentration to kill 90% of tested insects
LD_50_	Lethal Doses to kill 50% of tested insects
*r*	Intrinsic rate of increase
*R*_0_	Net reproductive rate
RH	Relative humidity
RP	Relative potency
SE	Standard error
α-NA	α-naphthyl acetate
β-NA	β-naphthyl acetate
λ	Finite rate of increase
χ^2^	Chi-square value
